# Molecular Mechanism of Action of Antimalarial Benzoisothiazolones: Species-Selective Inhibitors of the *Plasmodium* spp. MEP Pathway enzyme, IspD

**DOI:** 10.1038/srep36777

**Published:** 2016-11-18

**Authors:** Kathryn E. Price, Christopher M. Armstrong, Leah S. Imlay, Dana M. Hodge, C. Pidathala, Natalie J. Roberts, Jooyoung Park, Marwa Mikati, Raman Sharma, Alexandre S. Lawrenson, Niraj H. Tolia, Neil G. Berry, Paul M. O’Neill, Audrey R. Odom John

**Affiliations:** 1 Department of Chemistry, University of Liverpool, Liverpool, L69 7ZD, UK; 2 Department of Pediatrics, Washington University School of Medicine, St. Louis, MO 63110, USA; 3 Department of Molecular Microbiology, Washington University School of Medicine, St. Louis, MO 63110, USA

## Abstract

The methylerythritol phosphate (MEP) pathway is an essential metabolic pathway found in malaria parasites, but absent in mammals, making it a highly attractive target for the discovery of novel and selective antimalarial therapies. Using high-throughput screening, we have identified 2-phenyl benzo[*d*]isothiazol-3(2*H*)-ones as species-selective inhibitors of *Plasmodium* spp. 2-*C*-methyl-_D_-erythritol-4-phosphate cytidyltransferase (IspD), the third catalytic enzyme of the MEP pathway. 2-Phenyl benzo[*d*]isothiazol-3(2*H*)-ones display nanomolar inhibitory activity against *P. falciparum* and *P. vivax* IspD and prevent the growth of *P. falciparum* in culture, with EC_50_ values below 400 nM. *In silico* modeling, along with enzymatic, genetic and crystallographic studies, have established a mechanism-of-action involving initial non-covalent recognition of inhibitors at the IspD binding site, followed by disulfide bond formation through attack of an active site cysteine residue on the benzo[*d*]isothiazol-3(2*H*)-one core. The species-selective inhibitory activity of these small molecules against *Plasmodium* spp. IspD and cultured parasites suggests they have potential as lead compounds in the pursuit of novel drugs to treat malaria.

As the search for new antimalarial compounds has intensified, isoprenoid biosynthesis has emerged as an essential metabolic process that is sensitive to chemical inhibition and is thus a prominent candidate for drug intervention against the malaria parasite^1^,^2^,^3^,^4^. Isoprenoids represent a diverse class of natural products, which are essential for many cellular functions, including protein prenylation and electron transport^5^,^6^. The biosynthesis of isoprenoids requires the production of two building blocks: the universal five-carbon precursors, isopentenyl pyrophosphate (IPP) and its isomer dimethylallyl pyrophosphate (DMAPP)^7^. Mammals synthesize IPP and DMAPP via the well-studied, coenzyme-A dependent, mevalonate (MVA) pathway^8^. In contrast, eubacteria and plastid-containing eukaryotes, including *Plasmodium* spp. parasites, utilize the methylerythritol phosphate (MEP) pathway to generate isoprenoid precursors (Fig. 1)^2^,^9^. As the MVA and MEP pathways evolved independently, these pathways remain chemically and enzymatically distinct, enabling parasite-specific inhibition with minimal risk of toxicity to human cells^10^.

Small molecule inhibitors have been described against many enzymes of the MEP pathway through a number of programs aimed to harness the commercial, agricultural, and clinical potential of MEP pathway inhibition^1^,^11^,^12^. To date, the best-characterized inhibitor of the MEP pathway is the phosphonic acid antibiotic, fosmidomycin (FSM), a potent *in vitro* inhibitor of 1-deoxy-_D_-xylulose 5-phosphate reductoisomerase (IspC), the first dedicated enzyme in the MEP pathway (Fig. 1)^13^. FSM has undergone Phase II clinical trials as a potential antimalarial chemotherapeutic in combination with clindamycin and piperaquine^14^ and has therefore demonstrated the validity and safety of targeting MEP pathway enzymes as an antimalarial strategy^15^.

The second dedicated enzyme of the MEP pathway is 2-*C*-methyl-_D_-erythritol-4-phosphate cytidyltransferase (IspD), which catalyzes the condensation of methylerythritol phosphate (MEP) with cytidine triphosphate (CTP) to generate 4-diphosphocytidyl-2-*C*-methyl-_D_-erythritol (CDP-ME) and inorganic pyrophosphate^7^,^16^. We and others have previously reported small molecule inhibitors of IspD (**1** and **2**, Fig. 2), confirming that IspD homologs are amenable to chemical inhibition^12^,^17^,^18^. These IspD inhibitors have generally shown high-micromolar activity, although some herbicidal *A. thaliana* species-selective nanomolar templates have also been identified (**3a** and **3b**, Fig. 2). More recently, a chemical rescue screen identified **4** (1*R*,3*S*-MMV008138) from the Medicines for Malaria Venture (MMV) “malaria box” as a potent inhibitor of *P. falciparum* IspD (*Pf*IspD), showing nanomolar inhibition of recombinant enzyme activity and sub-micromolar inhibition of parasite growth (Fig. 2)^19^,^20^,^21^,^22^.

Here we report the discovery of a new series of antimalarial IspD inhibitors and determination of their mechanism of action. We have employed a combined approach of chemoinformatics and high-throughput enzymatic screening to identify a new, chemically distinct series of IspD enzyme inhibitors that inhibit *P. falciparum* growth through disruption of isoprenoid biosynthesis in the *Plasmodium* parasite. Following chemotype identification, we have further developed this series, building structure-activity relationship around the chosen chemical motif and improving drug-like inhibitor properties. Finally, we have employed a combination of molecular modelling studies, site-directed mutagenesis and structural elucidation to determine the molecular mechanism by which these compounds achieve enzyme inhibition. This work has identified a series of compounds that are chemically tractable, possess drug-like properties, and show significant promise for development as novel and potent antimalarial chemotherapies.

## Results

### Identification and optimization of benzoisothiazolone *Pf*IspD inhibitors

A high-throughput screen (HTS) was developed to identify inhibitors of purified recombinant *Pf*IspD. An assay protocol and chemoinformatics strategy (Methods: Chemoinformatics strategy) suitable for HTS were optimized and validated for screening with the Z’ ranging from 0.85 to 0.95^23^. With active compounds defined as displaying an *Pf*IspD IC_50_ of <20 μM, a total of 208 hit compounds were identified from the HTS study, comprising over 10 structural chemotypes. Significantly, the 2-phenyl benzo[*d*]isothiazol-3(2*H*)-one (BITZ) chemotype was repeatedly identified within a number of hit structures, including **7**, which exhibited a *Pf*IspD IC_50_ value of 450 ± 79 nM (Table 1).

**Figure 3:**
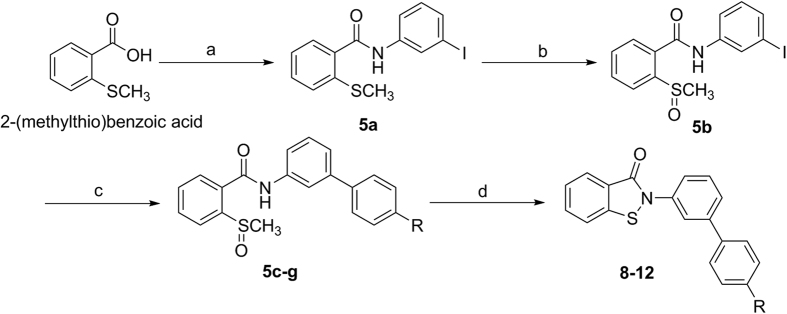
General synthetic strategy for inhibitors 8–12. (**a**) SOCl_2_ (3.0 eq), toluene, DMF, reflux, 2–3 hours: then 3-iodoaniline (1.2 eq), Et_3_N (2.0 eq), THF, 10 minutes at 0 °C, 20 hours at 22 °C; 98% yield. (**b**) NaIO_4_ in H_2_O (1.6 eq), MeOH, 50 °C, 4–5 hours; 96% yield. (**c**) Phenyl boronic acid (1.2 eq), K_2_CO_3_ (3.3 eq), [Pd(PPh_3_)_4_] (0.025 eq), H_2_O:THF (1:2), 80 °C, 18–22 hours; 80–87% yield. (**d**) SOCl_2_ (1.3 eq), DCM, 50 °C, 1.5 hours; 35–74% yield.

A number of analogs, based around **7**, were synthesized to explore the structure-activity relationship around the BITZ chemotype and analyze the nature and tolerance of the IspD active site. The bi-aryl BITZ template was selected to enable structural and chemical modification to the C and D rings adjacent to the inhibitor core (**8**–**13**, Table 1) with the aims of varying lipophilicity, improving solubility, and introducing functionality to enhance potency at *Pf*IspD. A representative four-step synthesis of target compounds is shown (Fig. 3) involving an acid-chloride mediated amide coupling of 2-(methylthio) benzoic acid and 3-iodoaniline, generating a (methyl-thio)benzamide, **5a**^24^. Oxidation of **5a** to the corresponding sulfoxide, **5b**^24^, followed by Suzuki-Miyaura cross-coupling reactions^25^, using a range of *para*-substituted phenyl boronic acids, was employed to generate intermediates **5c**–**g**. The methylsulfinyl benzamides, **5c**–**g**, were cyclized using a thionyl chloride-mediated ring closure^26^ to give selected inhibitors **8**–**12**. Inhibitor **13** was generated using an alternative three-step synthesis where a stable and isolatable acid chloride was employed for the generation of the core BITZ motif (Supplementary Fig. F1).

**Figure 4:**
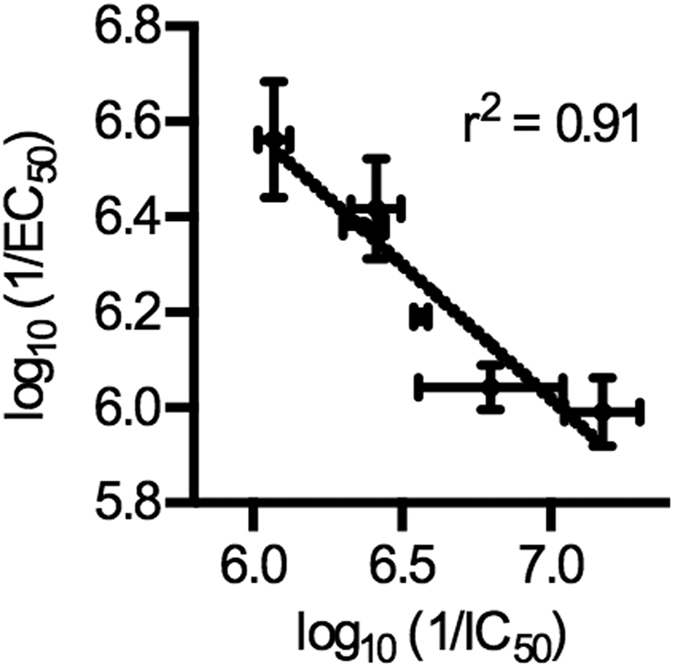
Correlation of enzyme *Pf*IspD enzymatic inhibition and anti-parasitic activity for inhibitors 8–13. Dose-dependent inhibition of enzyme activity and *P. falciparum* parasite growth was measured for 8–13 (Table 1) and half-maximal inhibitory concentrations determined prior to least-squares linear regression analysis (coefficient of determination, r^2^ = 0.91; GraphPad Prism). Mean values given with SEM; n ≥ 3.

Several compounds demonstrated low micromolar to nanomolar activity against *P. falciparum* and *P. vivax* IspD enzymes (Table 1), highlighting both the efficacy of these compounds and their potential to act as broad spectrum antimalarial agents. Overall, the series **8**–**13** demonstrated good correlation between *Pf*IspD enzyme inhibition and inhibition of parasite growth (r^2^ = 0.91, Fig. 4).

Given the ongoing and widespread issues of drug resistance to *P. falciparum*, it is critical that existing drug-resistant parasites are not cross-resistant to novel therapies in development. Therefore, we evaluated the antimalarial potency of BITZ compounds against a series of lab-adapted field isolates of *P. falciparum*, including strains resistant to chloroquine, mefloquine and artemisinin. We find that **8** effectively inhibits the growth of three lines of drug-resistant *P. falciparum* parasites (Table 2). These studies demonstrate the promise of BITZ compounds to inhibit parasite growth in *Plasmodium* strains with acquired resistance against current antimalarial drugs.

In optimising BITZ compounds, it was noted that *meta*-aryl functionalization across the C-ring consistently gave good levels of enzyme inhibition and that this substitution pattern is required to maintain potent *Pf*IspD inhibitory activity. In comparison, *para*-substitution across the C-ring lowered activity for series analogs, highlighting the importance of active site-inhibitor shape complementarity (Supplementary Table T1). *Para*-substitution within the D-ring, using electron releasing or withdrawing groups (**8**–**10**) was well tolerated, with *Pf*IspD IC_50_ values as low as 73 ± 20 nM and parasite EC_50_ of 1.1 ± 0.16 μM, with *P. vivax* IspD (*Pv*IspD) IC_50_ values of 57 ± 13 nM for the *para*-chloro analog **9**. In order to enhance aqueous solubility, methylene-linked polar heterocyclic groups were incorporated, exemplified by **11**–**13**. Inhibitor **11** showed a 100-fold increase in predicted aqueous solubility compared to **9**, a reduction in C*log*D from 4.98 to 3.80, and displayed nanomolar inhibitory activity against *Pf*IspD, *Pv*IspD, and cultured *P. falciparum*, demonstrating that inhibitor activity of this series can be maintained whilst enhancing drug-like properties.

### BITZ compounds inhibit isoprenoid metabolism in malaria parasites

**Figure 5:**
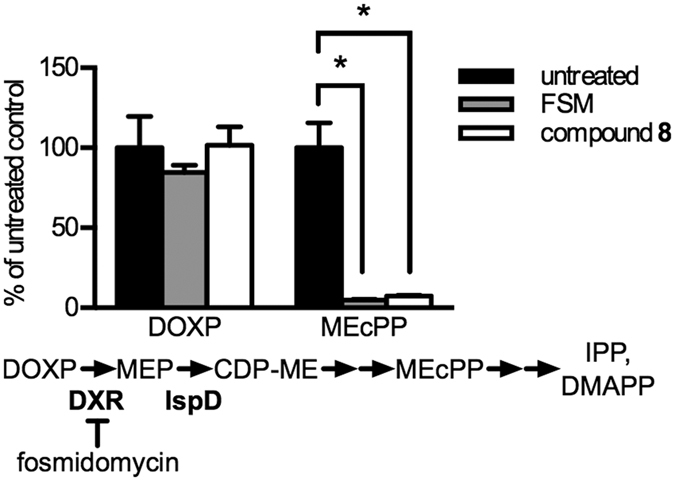
BITZ compounds inhibit the MEP pathway in *P. falciparum*. *P. falciparum* cultures were treated with 5 μM FSM or 3 μM 8 for 10 hours. Levels of the MEP pathway metabolites, DOXP and MEcPP, were measured using LC-MS/MS and compared to the levels in untreated parasites. Mean and standard error values from 3 independent experiments displayed. Asterisks (*) indicate significance threshold (alpha) = 0.05. Significant decreases in MEcPP levels were observed under treatment with both FSM (*p* = 0.0038) and compound 8 (*p* = 0.0042).

To establish that anti-parasitic activity was the result of methylerythritol phosphate (MEP) pathway inhibition, we performed targeted metabolic profiling of MEP pathway intermediates in parasites treated with our BITZ IspD-inhibiting compounds. Early ring-stage cultures of *P. falciparum* were treated with compound concentrations at ~5 times the IC_50_ of **8** for 10 hours and MEP pathway intermediates were quantified by LC-MS/MS analysis as described elsewhere^27^. Similar to the established MEP inhibitor, FSM, **8** produced a significant decrease in MEcPP levels (*p* = 0.004: Fig. 5); however, levels of the upstream metabolite, DOXP, were not affected by treatment with **8** (*p* = 0.94: Fig. 5). These results mirror those seen under comparable treatment with the established IspC inhibitor, FSM^19^, and therefore confirm that BITZ compounds inhibit the MEP pathway at levels known to affect asexual parasite growth.

In malaria parasites, supplementation with the isoprenoid precursor, IPP, has been demonstrated to rescue MEP pathway dysfunction. For example, IPP supplementation has been shown to allow parasite growth during treatment with either the DXR inhibitor, fosmidomycin (FSM), or the IspD inhibitor, 1*R*,3*S*-MMV008138^20^,^21^,^28^. In order to determine whether BITZ inhibition was specific to activity against the MEP pathway, we tested for IPP rescue during treatment with compound **8**. No rescue was observed (*p* = 0.75; Supplementary Table T2). The observed correlation between enzymatic inhibition and parasite growth inhibition (Fig. 4), as well as metabolic analysis (Fig. 5), suggest that growth inhibition under treatment with BITZ compounds involves inhibition of the IspD enzyme. However, this lack of rescue by IPP supplementation suggests that off-target activity also contributes to parasite growth inhibition.

### BITZ compounds inhibit IspD enzymes through covalent active site modification

**Figure 6:**
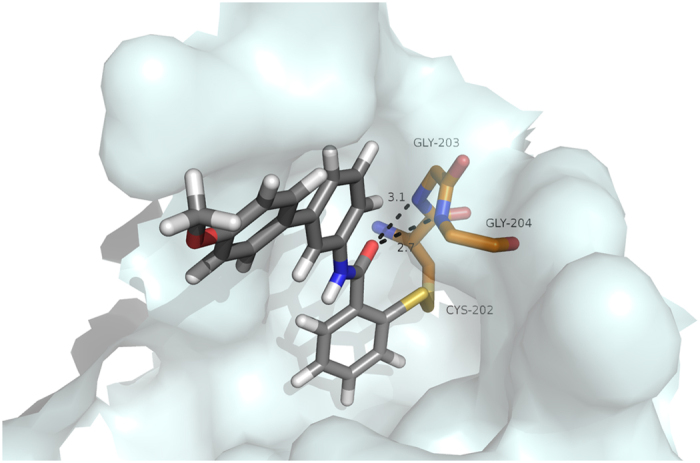
Inhibitor 8 modeled in the active site of *Pf*IspD homology model, covalently bound at Cys-202. *Pf*IspD active site rendered as a white surface. Residues predicted to interact with inhibitors (Gly-203 and Gly-204, hydrogen bonds; Cys-202, disulfide adduct) are depicted as sticks (carbon, orange; oxygen, red; nitrogen, blue; sulfur, yellow). 8 displayed as sticks (carbon, grey; oxygen, red; nitrogen, blue; sulfur, yellow). Hydrogen bonds depicted as black, dotted lines (predicted hydrogen bond distance in Ångstroms). Image created using PyMOL Molecular Graphics System, Version 1.5.0.4, Schrodinger, LLC.

**Figure 7:**
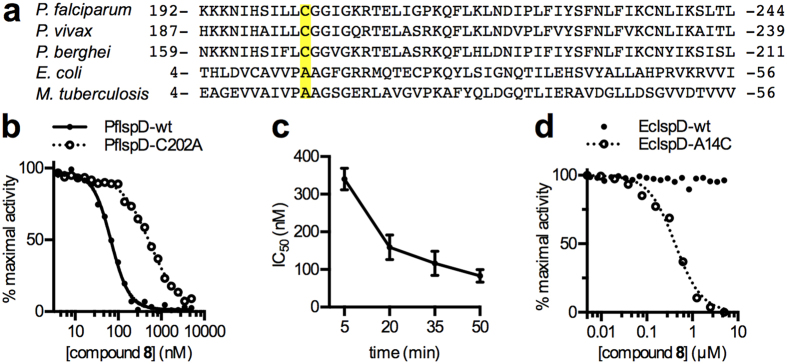
BITZ compounds inhibit *Pf*IspD through disulfide bond formation. (**a**) Alignment of IspD homologs: active site Cys residue is conserved in *Plasmodium* spp. but is absent in bacterial orthologs that are insensitive to BITZ inhibitors. (**b**) Dose-dependent inhibition of purified recombinant *Pf*IspD-wt compared to *Pf*IspD-C202A on treatment with 8. Representative data shown; n ≥ 3. (**c**) Time-dependent changes in inhibition of recombinant *Pf*IspD-wt enzyme activity following treatment with 8. Mean and SEM displayed; n ≥ 4 for each data point. (**d**) Dose-dependent inhibition of recombinant *Ec*IspD-wt compared to *Ec*IspD-A14C by 8. Representative data shown; n ≥ 3.

To gain insight into the mechanism of enzyme inhibition, we developed a *Pf*IspD homology model^19^ based on an *E. coli* IspD (*Ec*IspD) protein structure (PDB ID: 1I52^29^). Molecular docking of **8** clearly indicates that the inhibitor binds within the CTP substrate-binding pocket, placing the BITZ warhead proximal to the *Pf*IspD active site cysteine-202 (Cys-202) residue. We therefore predicted that the thiol moiety of Cys-202 would likely react with the sulfur heteroatom of BITZ inhibitors, forming a covalent disulfide adduct, while the carbonyl oxygen would be within hydrogen bonding distance of the backbone nitrogen atoms of Gly-203 and Gly-204 (Fig. 6). Formation of the covalent adduct would occlude the active site, preventing natural substrate binding and render the *Pf*IspD enzyme inactive. The active site Cys-202 residue is conserved in *Plasmodium* spp. IspD enzymes but not in bacterial homologs (Fig. 7a). BITZ compounds are found to be ineffective against *Ec*IspD enzyme activity (Fig. 7d), thus supporting the proposed involvement of and specific requirement for this Cys-202 residue, which is not present in the *E. coli* enzyme.

Our modelling studies predicted that mutation of Cys-202 to an alanine (Ala) residue would decrease the sensitivity of *Pf*IspD to this BITZ compound class. Indeed, introducing this *Pf*IspD mutation resulted in a 6-fold decrease in sensitivity: [*Pf*IspD-wt IC_50_ = 81 ± 13 nM, while *Pf*IspD-C202A IC_50_ = 470 ± 39 nM] (Fig. 7b) following prolonged (50 min.) incubations with **8**. This clear sensitivity change underlines the importance of Cys-202 for inhibitor activity, but may reflect both a loss of covalent bond formation and a change in compound fit within the active site following introduction of this mutation to the inhibitor binding region.

Covalent inhibition is often marked by an increase in the apparent potency of an inhibitor over time, as irreversible associations are gradually made between the inhibitor and enzyme. To evaluate this premise with respect to our proposed mechanism of enzyme inhibition, *Pf*IspD and various concentrations of **8** were pre-incubated prior to measurement of enzyme activity. As expected for covalent inhibition, the potency of **8** increased over time (*Pf*IspD-wt IC_50_ = 330 ± 57 nM at five min. and 81 ± 13 nM at 50 min.; *p* = 0.0007; Fig. 7c) whereas no such substantial or significant change in *Pf*IspD-C202A inhibition was observed over the same time period (*Pf*IspD-C202A IC_50_ data of 860 ± 220 nM at five min. and 470 ± 39 nM at 50 min.; *p* = 0.32). These results highlight the importance of the proposed covalent interactions with Cys-202 during inhibition of *Pf*IspD by BITZ inhibitors.

**Figure 8:**
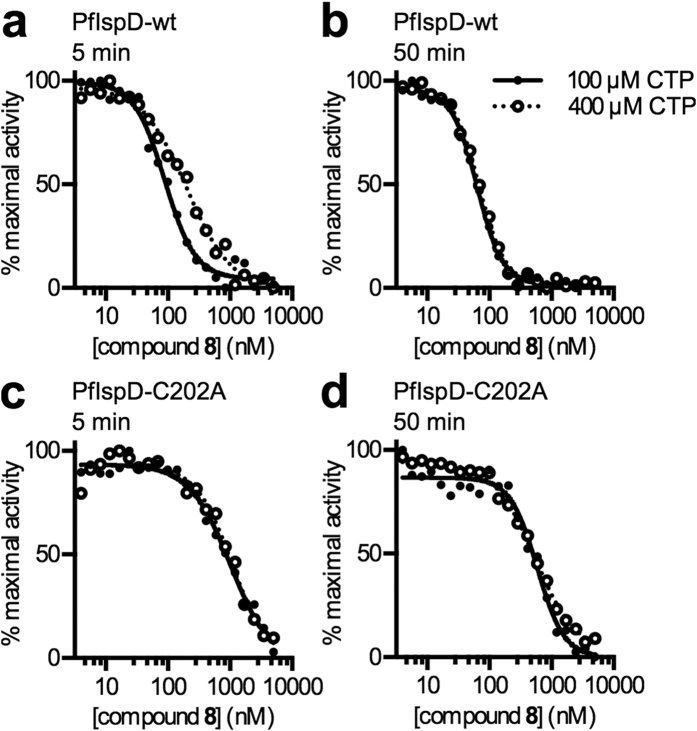
Inhibition of recombinant *Pf*IspD enzymes. (**a**) Treatment of *Pf*IspD-wt with 8 at varying CTP concentrations shows the concentration of CTP substrate affects the strength of inhibition at early time points. Five min. IC_50_ values gave 120 ± 17 nM at 100 μM CTP and 330 ± 57 nM at 400 μM CTP; *p* = 0.04. Representative data shown; n ≥ 3. (**b**) Treatment of *Pf*IspD-wt with 8 at varying CTP concentrations shows the concentration of CTP substrate does not affect the strength of inhibition following extended incubation. 50 min. IC_50_ values gave 49 ± 7.6 nM at 100 μM CTP and 81 ± 13 nM at 400 μM CTP; *p* = 0.17. Representative data shown; n ≥ 3. (**c**) Treatment of *Pf*IspD-C202A with 8 at varying CTP concentrations shows the concentration of CTP substrate does not affect the strength of inhibition at early time points. Five min. IC_50_ values gave 660 ± 130 nM at 100 μM CTP and 860 ± 120 nM at 400 μM CTP; *p* = 0.32. Representative data shown; n ≥ 3. (**d**) Treatment of *Pf*IspD-C202A with 8 at varying CTP concentrations shows the concentration of the CTP substrate does not affect the strength of inhibition following extended incubation. 50 min. IC_50_ values gave 414 ± 86 nM at 100 μM CTP and 470 ± 39 nM at 400 μM CTP; *p* = 0.57. Representative data shown; n ≥ 3.

Once an inhibitor-adduct has formed, increasing substrate concentration should not relieve covalent enzyme inhibition. Consistent with this model, the susceptibility of *Pf*IspD-wt to inhibition by **8**, following prolonged incubation, was not substantially affected by CTP substrate concentration (Fig. 8b). In contrast, inhibition by **8** was somewhat relieved under higher CTP concentrations over shorter incubation periods, as is typically observed for reversible competitive inhibitors (Fig. 8a). Together, these observations are consistent with relatively slow inhibitor-enzyme adduct formation within the CTP binding site, as predicted by our molecular docking model. Inhibition of *Pf*IspD-C202A activity was not significantly affected by CTP concentrations over short or long incubations with **8** (Fig. 8c,d), demonstrating that the CTP-site binding does not substantially contribute to inhibition of mutant *Pf*IspD-C202A and suggests an independent mode of inhibition of *Pf*IspD-C202A.

While both *P. falciparum* and *P. vivax* IspD homologs possess an active site Cys-202 residue, the corresponding residue in bacterial IspD homologs is alanine (Ala) (Fig. 7a). In contrast to the inhibitory IspD activity shown by BITZ analogues at *P. falciparum* and *P. vivax* IspD homologs (Table 1), we found that recombinant *Ec*IspD was highly resistant to inhibition by **8** (Fig. 7d). Therefore, to confirm the critical role of active site Cys residues in inhibition, we generated a variant *Ec*IspD protein, in which the corresponding Ala residue was substituted with a Cys residue by introduction of the A14C mutation. *Ec*IspD-A14C was rendered sensitive to **8**, with an IC_50_ of 440 ± 48 nM (Fig. 7d). *Ec*IspD-A14C was crystallized in the presence of 5 mM of **8** (Supplementary Table T3), providing supportive structural data. While electron density corresponding to **8** was not observed, the resulting crystal structure revealed formation of a novel intramolecular bond, leading to occlusion of the CTP binding site, thus providing modest additional evidence for a temporary covalent interaction between the inhibitor and the Cys-14 residue (Supplementary Fig. F2).

## Discussion

The non-mevalonate (MEP) pathway of isoprenoid biosynthesis is highly desirable as a target for antimalarial drug development, as the parasite-specific enzymes of this pathway do not have mammalian homologs. Since isoprenoid biosynthesis contributes to a number of essential cellular functions, MEP pathway function is required for asexual replication of *Plasmodium* spp. parasites. Despite the promise of new MEP pathway-targeting agents, few studies have explored the potential of targeting the second-dedicated enzyme of this pathway, 2-*C*-methyl-_D_-erythritol-4-phosphate cytidyltransferase (IspD), for the purpose of antimalarial chemotherapy. We have previously established that the *ISPD* gene of *P. falciparum* is resistant to genetic disruption and that the *Pf*IspD enzyme is amenable to small molecule inhibition^19^. We now report a new, structurally distinct class of *Pf*IspD 2-phenyl benzo[*d*]isothiazol-3(2*H*)-one (BITZ) inhibitors with antimalarial activity, which were identified through a target-based high-throughput study.

We find that BITZ compounds inhibit IspD enzymatic activity of both *P. falciparum* and *P. vivax* and that the IspD-inhibitory activity and antimalarial efficacy of these compounds are correlated (Fig. 4). Furthermore, we find that treatment of *P. falciparum* with growth-inhibitory concentrations of BITZ compounds is sufficient to disrupt *de novo* production of isoprenoid precursors. These findings strongly indicate that BITZ compounds inhibit parasite growth, at least in part, through inhibition of cellular IspD, as predicted. Importantly, parasite strains resistant to current generation antimalarials do not possess cross-resistance to BITZ compounds.

Our studies staunchly suggest that these novel IspD-inhibiting BITZ compounds have a distinctive mechanism of action that relies on the formation of a covalent enzyme-inhibitor adduct, with several lines of evidence supporting this mechanism of inhibition. First, BITZ inhibitors are competitive with CTP during short incubation periods but enzymatic inhibition cannot be out-competed following prolonged incubation of *Pf*IspD with the inhibitor, suggesting that a covalent bond is likely formed. Importantly, molecular modelling implicates a specific residue, Cys-202, as a likely site of enzyme modification by BITZ compounds. Supporting this model, we find that mutation of Cys-202 to an alanine residue confers BITZ resistance to recombinant *Pf*IspD enzyme (Fig. 7). In addition, we find that the bacterial IspD enzyme, *Ec*IspD, which possesses an alanine at the analogous position to Cys-202, is insensitive to BITZ compounds, but can be rendered sensitive via replacement of the alanine residue with cysteine. The proposed mechanism of enzyme inhibition thus provides an explanation for the marked species-selectivity of BITZ compounds and their inhibitory activity amongst IspD homologs, despite the overall sequence homology across this group of enzymes.

The demonstrated cysteine reactivity of our BITZ compound series suggests that development of this series may be hampered by possible polypharmacology. However, several recent reviews have noted that covalent mechanisms of action are prevalent among successful drugs^30^,^31^. Indeed, aspirin, also a covalent modifier, is one of the most widely used drug in the world. The polypharmacology of bioactive molecules^32^,^33^ and approved drugs^34^ has similarly been studied and is consistent with the concept that safe and effective drugs are invariably active at many targets. One such study calculates that, on average, approved therapeutics are active against approximately seven different molecular targets^35^. Since rescue with the isoprenoid precursor, IPP, is not observed, it is apparent that parasite growth inhibition by our BITZ compound series does not result solely from activity against IspD. Further studies may shed light on additional targets. Certainly continued vigilance will be required as BITZ compounds and their derivatives progress in development, with continued monitoring for potential promiscuous and off-target reactivity that may lead to toxicity. However, it should be noted that, similar BITZ chemotypes have demonstrated successful medicinal chemistry optimisation^36^,^37^,^38^,^39^,^40^,^41^,^42^. Recently, a molecule containing a BITZ moiety has begun Phase II clinical trials as an antiviral agent, providing important evidence that the potential reactivity of the BITZ moiety alone is not incompatible with a safe therapeutic profile^43^.

In conclusion, we have identified novel *Pf*IspD 2-phenyl benzo[*d*]isothiazol-3(2*H*)-one inhibitors, initiated from a HTS study. Following discovery of **7**, several structural analogs were generated around the identified BITZ motif which display increased potency, both enzymatically and phenotypically, compared to the initial hit. We have shown that the BITZ motif and corresponding analogues act as potent *Pf*IspD inhibitors and offer potential as a broad spectrum antimalarial agents. In addition, we have demonstrated that these compounds also express inhibitory activity against *P. falciparum* parasite strains which show drug resistance to existing antimalarial therapies. Our studies clearly define the mechanism of inhibition of the BITZ chemotype and corresponding analogues, with selectivity for *Pf*IspD relying on the presence of an active site Cys residue. A mechanism of inhibition has been deciphered whereby a disulfide adduct is formed between *Pf*IspD Cys-202 and the electrophilic sulfur atom of the BITZ inhibitor core. The next stages of hit development will focus on improving the efficacy, solubility, and PK properties of the most potent *Pf*IspD inhibitors. The potential promiscuity of the BITZ warhead and possible binding events at additional protein contacts will also require careful consideration. However, as interest in the covalent modification of drug targets within drug design increases, similar techniques and experiments to those outlined here may be used to probe other identified Cys modifiers which may be capable of enacting this mode of covalent modification. As more researchers study the MEP pathway and screen for inhibitors of its different enzymes, expectations dictate that it will become a recognized target for the treatment of not just malaria, but also tuberculosis and other bacterial pathogens.

## Methods

### Chemoinformatics strategy

A chemoinformatics strategy was employed to identify possible inhibitors using known IspD substrates and inhibitors, metal binding moieties, and biphosphate isosteres as query molecules for performing similarity and scaffold-hopping database searches^44^. A number of algorithms were applied, including molecular fingerprints^45^, turbo similarity^46^, principal component analysis, Bayesian modelling^47^, and machine learning to select ~10,000 compounds for screening. The selected structures were chosen from a commercial library of ~500,000 compounds (BioFocus DPI) that were predicted to possess favorable absorption, distribution, metabolism, excretion, and toxicity characteristics^48^.

### Molecular Modelling

A homology model of *Pf*IspD was constructed using the PHYRE online homology modelling program^49^. *Pf*IspD primary sequence Q8I273 was obtained from UNIPROT (http://www.uniprot.org/, accessed 10/12/14). A number of protein alignments and homology models were constructed by PHYRE, and the model with 98.82% confidence was selected, which was based on an *E. coli* IspD structure (PDB accession code 1I52)^29^. 1I52 is a 1.50-Å resolution crystal structure of *E. coli* IspD, complexed with CDP-ME in the active site. The structure of the model was validated using the WHATIF web interface^50^. **8** and other BITZ inhibitors were modelled *in silico* using the homology model described above in order to visualize the interactions between each analogue and the active site. Using GOLD, protons were added and docking was performed with default parameters, except that GoldScore was used and 50 docking poses were obtained for comparison and analysis. Covalent docking was performed using GOLD with the C-alpha atom of Cys-202 as the link atom to BITZ inhibitor **8**^51^.

### Purification of IspD proteins

Plasmids derived from pBG1861, containing wildtype or mutant alleles of *Plasmodium* spp. or *E. coli* IspD, were used to transform Artic Express (DE3) RIL *E. coli* cells (Stratagene). Cells were grown in LB broth with 100 μg/mL ampicillin at 37 °C and 200 rpm. During mid-logarithmic growth, cultures were cooled to 8 °C, and protein expression was induced with 1 mM IPTG for 16 hours.

After induction, cell pellets were lysed by sonication in lysis buffer (25 mM Tris pH 7.5, 250 mM NaCl, 1 mM MgCl_2_, 1 mM dithiothreitol (DTT), 20 mM imidazole, 10% glycerol, 0.1% Triton X-100, 200 μM PMSF, 1 mg/mL lysozyme, 0.3 U/mL benzonase nuclease (Novagen), and Roche Complete EDTA-free protease inhibitor). 6His-tagged IspD proteins were purified from soluble lysate over Ni-NTA resin (Goldbio). Beads were washed with 250 mM NaCl, 25 mM Tris pH 7.5, 1 mM MgCl_2_, 20 mM imidazole, and protein was eluted with 250 mM NaCl, 25 mM Tris pH 7.5, 1 mM MgCl_2_, 300 mM imidazole. Affinity purification of *Pv*IspD proceeded similarly to *Pf*IspD purifications, except that 0.1% Triton X-100 and 10% glycerol were not present in the lysis buffer. 10% glycerol was also absent from the lysis buffer used in purifying wildtype and A14C *E. coli* IspD, and lysis buffer used in purifying wildtype and C202A *Pf*IspD used in mechanistic studies (Figs 7 and 8).

Affinity-purified proteins were further purified over a HiLoad 16/60 Superdex 200 gel filtration column (GE Health Sciences), using an AKTA Explorer 100 FPLC (GE Health Sciences). FPLC buffer contained 250 mM NaCl, 25 mM Tris pH 7.5, 1 mM MgCl_2_. Fractions containing purified protein (>90% pure as evaluated by SDS-PAGE) were pooled and concentrated by centrifugation using Amicon Ultra-15 Centrifugal Filter Units (EMD Millipore). Concentrated protein was supplemented with 10% glycerol and 1 mM DTT (although DTT was omitted from wildtype and C202A *Pf*IspD preparations used in mechanism of action studies, (Figs 7 and 8), flash-frozen in liquid nitrogen, and stored at −80 °C. Protein concentration was measured using a BCA protein assay kit (Thermo Scientific).

### MEP metabolite profiling

Prior to treatment, parasites were synchronized by 1–2 treatments with 5% sorbitol until >8% parasitemia and >75% ring-stage cultures were achieved. At this point, cultures were treated 10 hours with 5 μM compound **8**, 5 μM fosmidomycin, or left untreated. Following treatment, parasite-infected erythrocytes were lysed with 0.1% saponin, washed in phosphate-buffered saline, and stored at −80°C until extraction and quantitative LC-MS/MS measurement of DOXP and MEcPP, as previously described. Values reflect the mean and standard error from ≥3 independent experiments and were compared using the Student’s t-test (two-tailed).

### Cloning of *Pf*IspD and orthologs

*P. falciparum*: The construct used for expression of the codon-optimized *Pf*IspD protein has been described previously^19^. To generate the *Pf*IspD variants containing the C202A mutation, N- and C-terminal fragments of the optimized *PfISPD* gene were amplified from the above vector using the following primers. C202A N-terminal fragment: T7 Fwd primer, 5′-TAATACGACTCACTATAGGG-3′, with C202A Rev primer, 5′-GTTTGCCAATACCGCCGGCTAGCAGGATACTATGAATG-3′; C202 C-terminal fragment, C202A Fwd primer, 5′-CATTCATAGTATCCTGCTAGCCGGCGGTATTGGCAAAC-3′, with T7 Rev primer, 5′-GCTAGTTATTGCTCAGCGG-3′. Amplicons containing the entire *PfISPD* gene were generated using *Pf*IspD Fwd and Rev primers, which introduce a 6-His tag, with N- and C-terminal fragments as template (*Pf*IspD Fwd primer: 5′-CTCACCACCACCACCACCATATGATGCACATCTACGATAATAATAA-3′; *Pf*IspD Rev: 5′-ATCCTATCTTACTCACTTATTTTGAGGAGTAGTAGAAT-3′). Following amplification, *PfISPD* variants were cloned into pBG1861 by ligation-independent cloning as previously described^19^,^52^, and verified by Sanger sequencing.

*P. vivax*: The construct used for expression of codon-optimized *Pv*IspD (*P. vivax* Sal-1; PVX_081425) has been previously described^19^.

*E. coli:* The construct used for expression of *Ec*IspD-wt has been previously described^27^. The A14C mutation was introduced by first generating N- and C-terminal gene fragments containing the mutation. These fragments were generated using the following primers: A14C N-terminal fragment: T7 Fwd primer, 5′-TAATACGACTCACTATAGGG-3′, with A14C Rev primer, 5′-CATTCGACGGCCAAATCCGGCACACGGAACCACGGCGCAAACATC-3′; A14C C-terminal fragment, A14C Fwd primer, 5′-GATGTTTGCGCCGTGGTTCCGTGTGCCGGATTTGGCCGTCGAATG-3′, with T7 Rev primer, 5′-GCTAGTTATTGCTCAGCGG-3′. Amplicons containing the entire *E. coli IspD* gene were generated using *Ec*IspD Fwd and Rev primers, which introduce a 6-His tag, with N- and C-terminal fragments as template (*Ec*IspD Fwd primer: 5′-CTCACCACCACCACCACCATATGATGGCAACCACTCATTTGG-3′; *Ec*IspD Rev primer: 5′-ATCCTATCTTACTCACTTATTTGTATTCTCCTGATGG-3′). Following amplification, *E. coli A14C-IspD* was cloned into pBG1861 by ligation-independent cloning as previously described^52^, and verified by Sanger sequencing.

### IspD assay conditions

Phosphate released by IspD was quantified using the EnzChek Phosphate Assay Kit (Invitrogen, Life Technologies), as previously described^27^. 2-Amino-6-mercapto-7-methylpurine riboside (MESG) and purine nucleoside phosphorylase (PNP) were diluted and stored according to the manufacturer’s instructions. Final concentrations of reagents were as follows: 100 mM NaCl, 25 mM Tris pH 7.5, 7.5 mM MgCl_2_, 1 U/ml PNP, 0.1 U/mL yeast inorganic pyrophosphatase (New England Biolabs). Unless otherwise specified, IC_50_ assays contained 50 nM enzyme, 400 μM CTP (Sigma), 200 μM MEP (Echelon Biosciences), 2% DMSO (vehicle), and 200 μM MESG. Reactions were performed in 50 μL final volumes in 96-well clear flat bottom plates. In general, enzymes, buffers, and inhibitors were pre-warmed approximately 15 minutes at 37 °C, and reactions were then initiated by addition of the MEP substrate. Timed inhibitor **8** IC_50_ assays (5, 20, 35, and 50 minute incubations) and comparisons between behavior at 100 μM and 400 μM CTP were initiated by the addition of the CTP substrate and contained 100 nM enzyme. Assays using *E. coli* IspD used 7.5 nM *Ec*IspD-wt or 75 nM *Ec*IspD-A14C. Absorbance at 360 nm was measured over time on a BMG POLARStar plate reader, preheated to 37 °C. Nonlinear regression analysis was performed using GraphPad Prism software. Values reflect the mean and standard error from ≥3 independent experimental and were compared using the Student’s t-test (two-tailed). Slopes of changing absorbance values were converted to (μM MEP) (μM enzyme)^−1^ s^−1^ using a phosphate standard curve.

### *P. falciparum* culture

Unless otherwise specified, *P. falciparum* parasites were cultured as previously described^6^,^53^, in a 2% suspension of human erythrocytes in RPMI-1640 medium (Sigma Aldrich) supplemented with 27 mM sodium bicarbonate, 11 mM glucose, 5 mM HEPES, 1 mM sodium pyruvate, 0.37 mM hypoxanthine, 0.01 mM thymidine, 10 μg ml^−1^ gentamicin and 0.5% Albumax (Life Technologies). Cultures were maintained in 5% O_2_/5% CO_2_/90% N_2_ at 37 °C. Parasite growth was monitored by microscopy of Giemsa-stained parasites. 3D7 strain parasites were obtained from the Malaria Research and Reference Reagent Resource Center, ATCC, Manassas, Virginia. D6 and 7G8 strains were obtained through BEI Resources Repository, NIAID, NIH: *Plasmodium falciparum*, Strain D6, MRA-285, contributed by D.E. Kyle; *Plasmodium falciparum*, Strain 7G8, MRA-152, contributed by David Walliker. The IPC 5202 strain was provided by the Malaria Research and Reference Reagent Resource Center (MR4) for distribution by BEI Resources NIAID, NIH: *Plasmodium falciparum*, Strain IPC 5202, also known as CAM3.IR539T; MRA-1240, contributed by Didier Ménard.

### Parasite sensitivity assays

Asynchronous cultures of *P. falciparum* strain 3D7 were diluted to 1% parasitemia and cultured at indicated concentrations of inhibitor compounds in 100 μL culture volumes. During rescue experiments, parasite growth medium was supplemented with 200 μM isopentenyl pyrophosphate (IPP; Echelon Biosciences). After 72 hours, parasite growth was quantified using PicoGreen dye (Invitrogen) to measure DNA content as previously described^27^. PicoGreen fluorescence was measured (485 nm excitation/528 nm emission) in a POLARStar Omega microplate reader (BMG Labtech). IC_50_ values were calculated by nonlinear regression analysis using GraphPad Prism software, and reflect the mean and standard error from ≥3 independent experiments. Values were compared using the Student’s t-test (two-tailed).

## Additional Information

**How to cite this article**: Price, K. E. *et al*. Molecular Mechanism of Action of Antimalarial Benzoisothiazolones: Species-Selective Inhibitors of the *Plasmodium* spp. MEP Pathway enzyme, IspD. *Sci. Rep.* **6**, 36777; doi: 10.1038/srep36777 (2016).

**Publisher’s note:** Springer Nature remains neutral with regard to jurisdictional claims in published maps and institutional affiliations.

## Supplementary Material

Supplementary Information

## Figures and Tables

**Figure 1:**
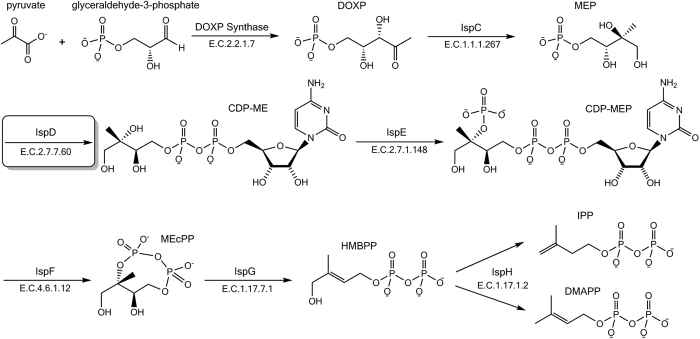
The non-mevalonate methylerythritol phosphate (MEP) pathway of isoprenoid biosynthesis. Chemical structures of the native substrates and products of each enzyme in the MEP pathway are depicted: 1-deoxy-_D_-xylulose-5-phosphate synthase (DOXP Synthase); 1-deoxy-_D_-xylulose-5-phosphate reductoisomerase (IspC); 2-*C*-methyl-_D_-erythritol-4-phosphate cytidyltransferase (IspD); 4-diphosphocytidyl-2*C*-methyl-_D_-erythritol kinase (IspE); 2-*C*-methyl-_D_-eryth-ritol-2,4-cyclodiphosphate synthase (IspF); 1-hydroxy-2-methyl-2-(*E*)-butenyl-4-diphosphate synthase (IspG); 4-hydroxy-3-methyl-2-(*E*)-butenyl-4-diphosphate reductase (IspH).

**Figure 2:**
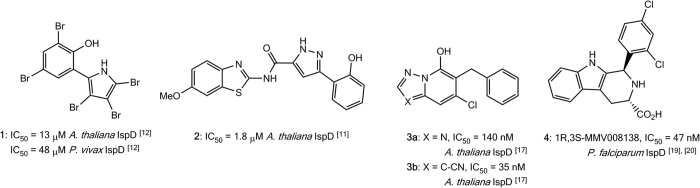
Known IspD inhibitors.

**Table 1:**
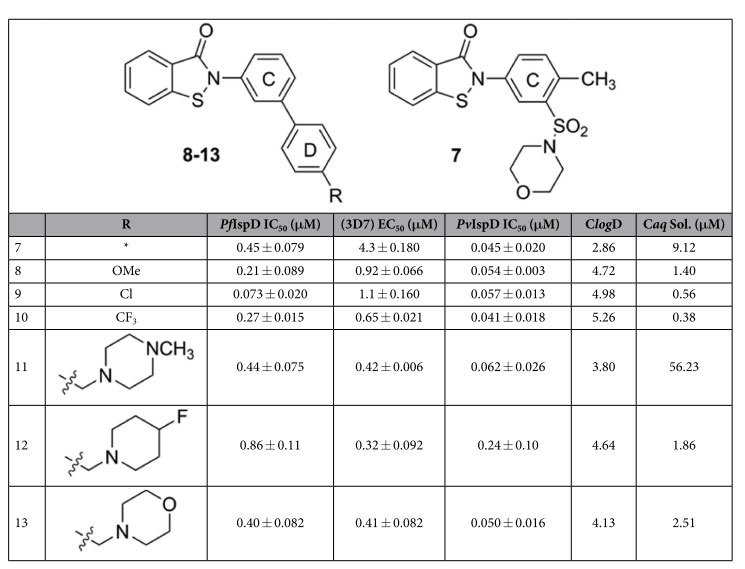
Chemical structures of 7–13 with corresponding inhibitory activity (mean and SEM; n ≥ 3) against *Pf*IspD, *P. falciparum* growth in culture (strain 3D7), and *Pv*IspD.

ClogD and Caq Sol. calculated using algorithms from AstraZeneca^54^,^55^.

**Table 2:** Inhibitory activity of 8 against drug-resistant *P. falciparum* parasites grown in culture (mean and SEM; ≥3).

*P. falciparum* strain	Whole Cell Growth Inhibition EC_50_ (μM)
3D7	0.92 ± 0.066
D6	0.80 ± 0.12
7G8	1.02 ± 0.25
IPC 5202	1.38 ± 0.19

Strains: 3D7, pan-sensitive; D6, mefloquine-resistant^56^; 7G8, multidrug (chloroquine)-resistant^57^; IPC 5202, artemisinin-resistant^58^.
