# Supplementary material for: Molecular Mechanism of Action of Antimalarial Benzoisothiazolones: Species-Selective Inhibitors of the Plasmodium spp. MEP Pathway enzyme, IspD
Source: Sci Rep. 2016 Nov 18;6:36777. doi: 10.1038/srep36777 (PMC5114681; doi:10.1038/srep36777)
Supplement: Supplementary Information [file srep36777-s1.pdf]

Supporting Information

Molecular Mechanism of Action of Antimalarial Benzoisothiazolones: Species-Selective Inhibitors of the *Plasmodium* spp. MEP Pathway enzyme, IspD.

Kathryn E. Price, Christopher M. Armstrong, Leah S. Imlay, Dana M. Hodge, C. Pidathala, Natalie J. Roberts, Jooyoung Park, Marwa Mikati, Raman Sharma, Alexandre S. Lawrenson, Niraj H. Tolia, Neil G. Berry, Paul M. O'Neill, and Audrey R. Odom.

Supporting Information

Table of Contents **Page No.**

Supplementary Tables

Table T1 S3
Table T2 S3-4
Table T3 S4

Supplementary Figures

Figure F1 S5
Figure F2 S6

Supplementary Data
Chemistry

Preparation of *N*-(3-iodphenyl)-2-methylthio)benzamide **5a** S7
Preparation of *N*-(3-iodphenyl)-2-methylsulfinyl)benzamide **5b** S7-8
General procedure for the preparation of biphenyl methylsulfinyl benzamides **5c-g** S8
Preparation of *N*-(4'-methoxy-[1,1'-biphenyl]-3-yl)-2-(methylsulfinyl) benzamide **5c** S8-9
Preparation of *N*-(4'-chloro-[1,1'-biphenyl]-3-yl)-2-(methylsulfinyl) benzamide **5d** S9
Preparation of 2-(methylsulfinyl)-*N*-(4'-(trifluoromethyl)-[1,1'-biphenyl]-3-yl) benzamide **5e** S9
Preparation of *N*-(4'-((4-methylpiperazin-1-yl)methyl)-[1,1'-biphenyl]-3-yl)-2-(methylsulfinyl) benzamide **5f** S9-10
Preparation of *N*-(4'-((4-fluoropiperidin-1-yl)methyl)-[1,1'-biphenyl]-3-yl)-2-(methylsulfinyl) benzamide **5g** S10
General procedure for the preparation of benzo[*d*]isothiazol-3(2*H*)-ones **8-12** S10-11
Preparation of 2-(4'-methoxy-[1,1'-biphenyl]-3-yl)benzo[*d*]isothiazol-3(2*H*)-one **8** S11
Preparation of 2-(4'-chloro-[1,1'-biphenyl]-3-yl)benzo[*d*]isothiazol-3(2*H*)-one **9** S11
Preparation of 2-(4'-(trifluoromethyl)-[1,1'-biphenyl]-3-yl)benzo[*d*]isothiazol-3(2*H*)-one **10** S11-12
Preparation of 2-(4'-((4-methylpiperazin-1-yl)methyl)-[1,1'-biphenyl]-3-yl) benzo[*d*]isothiazol-3(2*H*)-one **11** S12
Preparation of 2-(4'-((4-fluoropiperidin-1-yl)methyl)-[1,1'-biphenyl]-3-yl) benzo[*d*]isothiazol-3(2*H*)-one **12** S12

Preparation (4-(morpholinomethyl)phenyl)boronic acid **6a** S13
Preparation 4'-(morpholinomethyl)-[1,1'-biphenyl]-3-amine **6b** S13
Preparation 2-(chlorothio)-benzoyl chloride S13-44
Preparation of 2-(4'-(morpholinomethyl)-[1,1'-biphenyl]-3-yl) benzo[*d*]isothiazol-3(2*H*)-one **13** S14

Biology

Crystallization S14-15

References S15-16

Supplementary Tables

Table T1. Ligand docking scores of BITZ compounds with *para*-substitution across the C-ring in the *Pf*IspD homology model and inhibitory activity of the compounds against the *Pf*IspD enzyme (IC_{50}, mM).

Chemical Structure	Ligand Docking Score	HTS *Pf*IspD IC_{50} (mM)
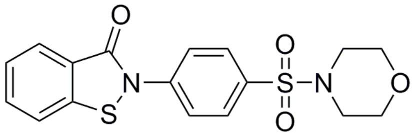	45 ± 1.0	> 40
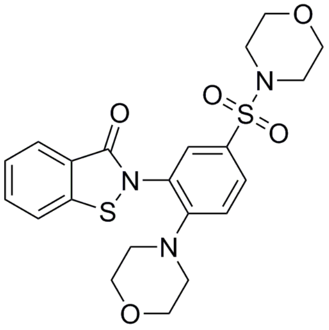	62 ± 2.0	0.379

Inhibitor structures were prepared for docking in Spartan'08 (Wavefunction Inc., Irvine, California, USA; 1991–2009). For inhibitor docking studies, GOLD 5.2 (CCDC Software Limited, Cambridge, UK)[1] was used to find potential binding poses in our own homology model of *Pf*IspD. Hydrogen atoms were added to the protein and the Cys-202 residue was used to define the ligand binding site as residues within 10 Å of Cα. Covalent docking was performed using Cα of Cys-202 as the link atom. Inhibitors were docked 25 times using the CHEMPLP fitness function. All other settings were left at default.

Table T2. Activity of compound 8 in the presence and absence of IPP supplementation.

Growth conditions	3D7 EC_{50} (μM)
no supplementation	0.92 ± 0.066
200 μM IPP	0.98 ± 0.24

Parasite growth inhibition assays were performed in the presence and absence of supplementation with the MEP pathway product and isoprenoid precursor, IPP

(mean and SEM, n ≥ 3). Although simultaneous control experiments demonstrated IPP rescue of fosmidomycin-treated parasites, we did not observe rescue of parasites treated with compound 8 (p = 0.75).

Table T3. Data collection and refinement statistics for co-crystallization of *Ec*IspD-A14C with inhibitor 8 at 5 mM.

	***Ec*IspD-A14C**
**Data collection**	
Space group	C2
Cell dimensions	
a, b, c, (Å)	94.68, 47.67, 53.95
α, β, γ (°)	90.00, 99.515, 90.00
Resolution (Å)*	20-1.60 (1.70-1.60)*
R_{meas}	7.3% (46.4%)
$I/\sigma I$	11.29 (2.56)
Completeness (%)	96.3% (98.2%)
Redundancy	3.52 (3.43)
**Refinement**	
Resolution (Å)	20-1.60
No. of reflections	30,303
R_{work}/R_{free}	17.46/19.98
No. of atoms	
Protein	1,775
Ca	1
Water	165
B-factors	
Protein	26.43
Ligands	18.16
Water	32.95
R.m.s. deviations	
Bond lengths (Å)	0.008
Bond angles (°)	1.246

Supplementary Figures

Figure F1. Synthetic strategy for inhibitor 13.

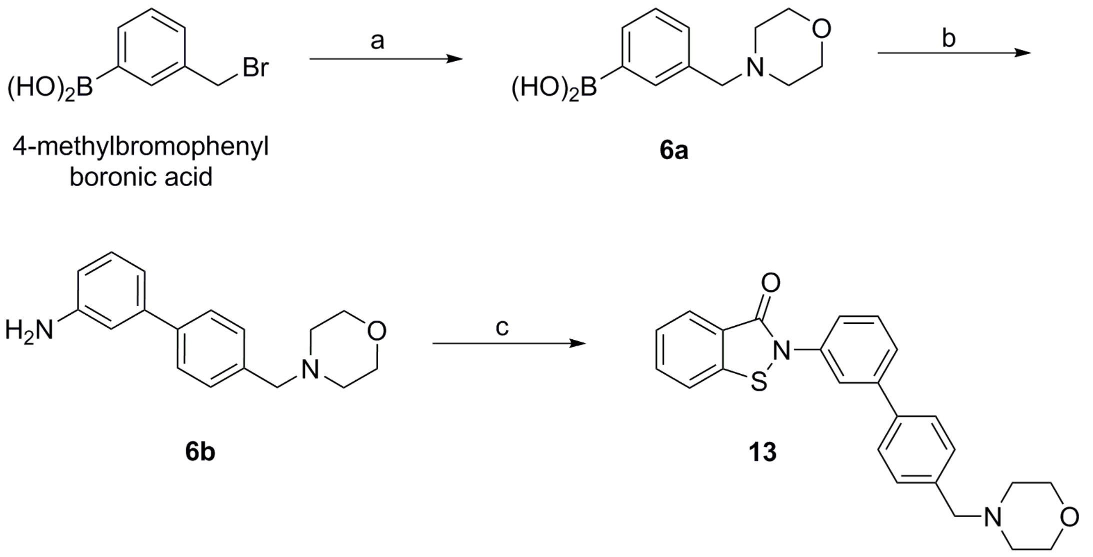

a) Morpholine (2.0 eq), DIPEA (2.5 eq), THF, rt, 18 hours; 87% yield.

b) K_2CO_3 (3.3 eq), [Pd(PPh_3)$_4$] (0.025 eq), 3-bromoaniline (0.8 eq), H_2O:THF (1:2), 80°C, 22 hours; 91% yield.

c) 2-(chlorothio)-benzoyl chloride (1.2 eq), Et_3N (3.3 eq) at 0°C in DCM, rt, 20 hours; 79% yield.

Nucleophilic displacement of a methylbromine by morpholine gave **6a**[2] which underwent a Suzuki-Miyaura cross-coupling reaction with 3-bromoaniline to generate the intermediate **6b**. This was cyclized with the stable and isolatable acid chloride, 2-(chlorothio)-benzoyl chloride[3] in a simultaneous amide coupling and ring closure mechanism to produce **13**.[4,5]

Figure F2. Inhibitor 8 induces disulfide-bridge formation, altering the *Ec*IspD-A14C active site.

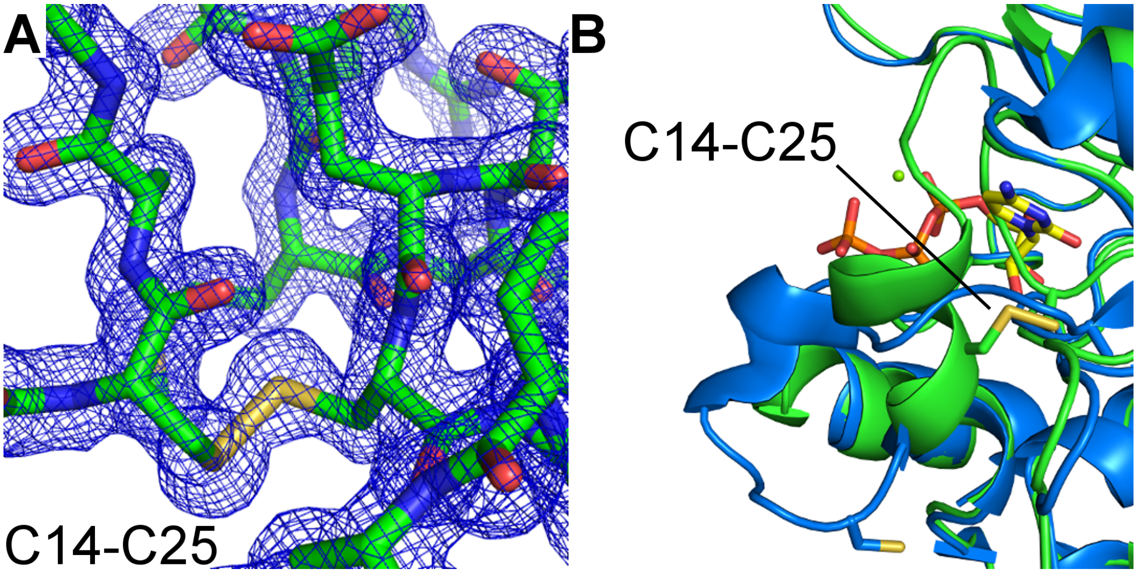

a) The 2fo-fc electron density map contoured at 1.0 σ for the disulfide bridge formed between Cys-14 and Cys-25.

b) The loop spanning residues 14-25 bind CTP in the 1I52 structure[6] (blue). Formation of the disulfide bridge in the enzyme-inhibitor complex between *Ec*IspD-A14C and compound 8 results in loop closure that occludes the CTP binding site (green).

*Ec*IspD-A14C was co-crystallized with 5 mM compound **8** (Supplementary Table T2), revealing a newly formed intramolecular disulfide bond between Cys-14 and Cys-25. Previous crystal structures show that in the absence of CTP, loop spanning residues14-25 are disordered and flexible in the *Ec*IspD protein (PDB ID: 1VGT[7], 1H3M[8], 3N9W[9]) whereas, in the presence of CTP (PDB ID: 1I52[6]), this loop is ordered and makes important contacts with CTP. In the mutant *Ec*IspD-A14C structure, the now-accessible disulfide bridge results in a closed conformation of the loop which occludes the CTP binding pocket (Supplementary Fig. F2). Altogether, these studies provide evidence that the *Pf*IspD enzyme is inhibited by BITZ compounds via covalent bond formation between the active site Cys-202 residue and the BITZ core.

Supplementary Data
Chemistry

Preparation of ***N*-(3-iodophenyl)-2-(methylthio) benzamide 5a**

To a stirring solution of 2-(methylthio)benzoic acid (13.8 mmol) in anhydrous toluene (25 ml) under a N_2 atmosphere, thionyl chloride (41 mmol, 3.0 eq) was added, followed by 3 drops of *N*,*N*-dimethylformamide; the solution was refluxed for 2-3 hours. Anhydrous toluene and excess thionyl chloride were removed under reduced pressure to give the acid chloride intermediate as a white solid. 3-Iodoaniline (16.4 mmol, 1.2 eq) was dissolved in anhydrous THF (10 ml/mmol), to which triethylamine (27.4 mmol, 2.0 eq) was added. The crude acid chloride was re-dissolved in anhydrous THF (10 ml/mmol) and added drop-wise to the amine and base solution over 10 minutes at 0°C. After a further 10 minutes, the solution was warmed to room temperature and stirred for 20 hours. After this time, the solvent was removed under reduced pressure; the resulting material diluted with water (30 ml) and the organic material extracted into EtOAc (3 x 30 ml). The combined organic extracts were washed with 5% HCl (50 ml), saturated aq. $NaHCO_3$ (50 ml) and brine (50 ml), dried over $MgSO_4$, filtered and concentrated under reduced pressure to give **5a** in 97.7% yield. **^{1}H NMR** (400 MHz, $CDCl_3$) δ 8.69 (s, 1H), 8.08 (s, 1H), 7.70 – 7.61 (m, 2H), 7.51 – 7.46 (m, 1H), 7.46 – 7.41 (m, 1H), 7.40 – 7.35 (m, 1H), 7.29 – 7.24 (m, 1H), 7.09 (t, *J* = 8.1 Hz, 1H) and 2.50 (s, 3H); **^{13}C NMR** (101 MHz, $CDCl_3$) δ 139.4, 137.3, 135.2, 133.9, 131.6, 130.9, 129.3, 120.0, 128.2, 126.0, 119.6, 115.5, 94.6 and 17.3; **ES+HRMS** *m/z* 391.9570 $[M+Na]^+$ $C_{14}H_{12}INOS$ requires 368.97.

Preparation of ***N*-(3-iodophenyl)-2-(methylsulfinyl) benzamide 5b**

A solution of sodium periodate (11.68 mmol, 1.6 eq) in water (30 ml) was added dropwise to **5a** (13.4 mmol) in MeOH (60 ml) at room temperature. The biphasic solution was heated to 50°C for 4-5 hours before the solvent was removed under reduced pressure. The crude precipitate was diluted with water, filtered through a sinter and washed with water before drying for 12 hours in a desiccator to give **5b** as a fine

brown powder in 96.3% yield. **^{1}H NMR** (500 MHz, DMSO) δ 10.67 (s, 1H, NH), 8.21 (t, *J* = 1.8 Hz, 1H), 8.16 (dd, *J* = 7.8, 1.3 Hz 1H), 7.99 (dd, *J* = 7.8, 1.0 Hz, 1H), 7.88 (td, *J* = 7.4, 1.3 Hz, 1H), 7.72 (ddd, *J* = 4.5, 2.8, 1.1 Hz, 2H), 7.51 (ddd, *J* = 7.8, 1.7, 1.0 Hz, 1H), 7.19 (t, *J* = 8.1 Hz, 1H), 2.83 (s, 3H); **^{13}C NMR** (101 MHz, DMSO) δ 165.1, 148.9, 140.5, 133.2, 132.9, 132.3, 131.3, 130.9, 128.9, 128.5, 124.2, 120.0, 94.9, 45.1; **ES+HRMS** *m/z* 407.9529 $[M+Na]^+$ $C_{14}H_{12}INO_2S$ requires 384.96.

Preparation of biphenyl methylsulfonyl benzamides 5c-g

General procedure (1)

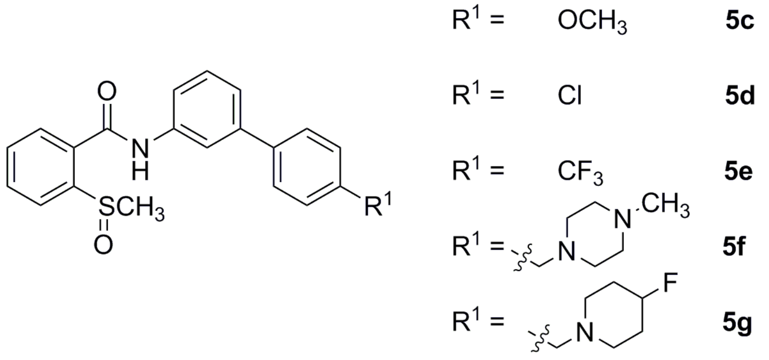

To a solution of K_2CO_3 (3.3 eq), **5b** (1.0 eq), and $Pd(PPh_3)_4$ (0.025 eq) in water (5 ml), the required phenyl boronic acid (1.2 eq) was added. The stirring solution was evacuated and purged with N_2 before adding anhydrous THF (10 ml) and heating to 80°C for 18-22 hours. After this time, the solution was quenched with 2N HCl (15 ml) and extracted into EtOAc (3 x 30 ml). The combined organic extracts were washed with brine (30 ml), dried over $MgSO_4$ and concentrated under reduced pressure. The crude products were purified by flash column chromatography.

Preparation of ***N*-(4'-methoxy-[1,1'-biphenyl]-3-yl)-2-(methylsulfinyl)benzamide 5c**

The general procedure **(1)** was followed using 4-(methoxy)-phenyl boronic acid to give **5c** as a cream-brown crystalline solid in 85.9% yield. **^{1}H NMR** (500 MHz, DMSO) δ 10.66 (s, 1H, NH), 8.17 (d, *J* = 7.8 Hz, 1H), 8.02 (d, *J* = 7.6 Hz, 1H), 7.98 (s, 1H), 7.89 (t, *J* = 7.6 Hz, 1H), 7.77 – 7.68 (m, 2H), 7.59 (d, *J* = 8.6 Hz, 2H), 7.44 (t, *J* = 7.8 Hz, 1H), 7.39 (d, *J* = 7.7 Hz, 1H), 7.06 (d, *J* = 8.6 Hz, 2H), 3.81 (s, 3H), 2.84 (s, 3H); **^{13}C NMR**

(101 MHz, DMSO) δ 165.2, 159.5, 148.8, 140.9, 139.6, 132.8, 132.8, 132.7, 130.9, 129.7, 128.5, 128.2 (2C), 124.12, 122.6, 119.2, 118.7, 114.9 (2C), 55.7, 45.1; **ES+HRMS** *m/z* 388.0974 $[M+Na]^+$ $C_{21}H_{19}NO_3S$ requires 365.11.

Preparation of ***N*-(4'-chloro-[1,1'-biphenyl]-3-yl)-2-(methylsulfinyl)benzamide 5d**

The general procedure **(1)** was followed using 4-(chloro)-phenyl boronic acid to give **5d** as a white solid in 83.4% yield. ^{1}**H NMR** (400 MHz, MeOD) δ 8.23 (dd, *J* = 8.0, 1.0 Hz, 1H), 8.05 – 8.00 (m, 1H), 7.98 (d, *J* = 1.7 Hz, 1H), 7.88 (td, *J* = 7.7, 1.2 Hz, 1H), 7.73 (td, *J* = 7.6, 1.2 Hz, 1H), 7.69 – 7.65 (m, 1H), 7.65 – 7.61 (m, 2H), 7.49 – 7.41 (m, 4H) and 2.98 (s, 3H); ^{13}**C NMR** (101 MHz, DMSO) δ 165.1, 148.7, 139.8, 139.6, 139.2, 132.9, 132.7, 132.5, 130.8, 129.9, 129.4, 128.8, 128.4, 124.0, 122.9, 120.1, 118.9 and 45.0; **ES+HRMS** *m/z* 392.0474 $[M+Na]^+$ $C_{20}H_{16}ClNO_2S$ requires 369.06.

Preparation of **2-(methylsulfinyl)-*N*-(4'-(trifluoromethyl)-[1,1'-biphenyl]-3-yl) benzamide 5e**

The general procedure **(1)** was followed using 4-(trifluoromethyl)-phenyl boronic acid to give **5e** as a cream solid in 86.8% yield. ^{1}**H NMR** (500 MHz, DMSO) δ 10.75 (s, 1H, NH), 8.18 (dd, *J* = 7.7, 1.0 Hz 1H), 8.12 – 8.10 (m, 1H), 8.03 (dd, *J* = 7.7, 1.0 Hz, 1H), 7.89 (ddd, *J* = 7.9, 7.4, 1.3 Hz, 1H), 7.87 (s, 4H), 7.84 – 7.79 (m, 1H), 7.74 (td, *J* = 7.5, 1.3 Hz, 1H), 7.54 – 7.52 (m, 2H), 2.84 (s, 3H); ^{13}**C NMR** (101 MHz, DMSO) δ 165.3, 148.9, 144.5, 139.8, 139.7, 132.8, 132.6, 130.9, 130.1, 128.5 (d, $^2J_{CF}$ = 31.8 Hz, 1C), 128.5, 127.9 (2C), 126.4 (d, $^3J_{CF}$ = 3.9 Hz, 2C), 124.8 (d, $^1J_{CF}$ = 271.9 Hz, 1C), 124.2, 123.4, 120.8, 119.4, 45.1; **ES+HRMS** *m/z* 426.0750 $[M+Na]^+$ $C_{21}H_{16}F_3NO_2S$ requires 403.09.

Preparation of ***N*-(4'-((4-methylpiperazin-1-yl)methyl)-[1,1'-biphenyl]-3-yl)-2-(methylsulfinyl) benzamide 5f**

The general procedure **(1)** was followed using 1-methyl-4-[4-(4,4,5,5-tetramethyl-1,3,2-dioxaborolan-2-yl)benzyl]piperazine to give **5f** as a clear pale yellow viscous oil in 85.9% yield. ^{1}**H NMR** (400 MHz, MeOD) δ 8.25 (d, *J* = 7.6 Hz, 1H), 8.05 (d, *J* = 7.6 Hz, 1H), 8.01 (s, 1H,), 7.90 (t, *J* = 7.4 Hz, 1H), 7.75 (t, *J* = 7.4 Hz, 1H,), 7.68-7.63 (m, 3H),

7.47-7.44(m, 4H), 3.62 (s, 2H), 2.99 (s, 3H), 2.69 (br s, 8H), 2.82-2.48 (s, 3H); **^{13}C NMR** (101 MHz, MeOD) δ 165.3, 147.0, 141.5, 139.7, 138.6, 136.3, 132.4, 132.4, 130.7, 129.8 (2C), 129.0, 127.9, 126.6 (2C), 123.6, 123.1, 119.5, 119.1, 61.9, 54.2 (2C), 51.9 (2C), 44.4, 43.8. **Elemental analysis** (%) found: C, 68.19; H, 6.57; N, 9.21; S, 6.26%. $C_{26}H_{29}N_3O_2S$ requires C, 69.77; H, 6.53; N, 9.39; S, 7.16%; **ES+HRMS** *m/z* 448.2063 $[M+H]^+$ $C_{26}H_{29}N_3O_2S$ requires 447.20.

Preparation of ***N*-(4'-((4-fluoropiperidin-1-yl) methyl)-[1,1'-biphenyl]-3-yl)-2-(methylsulfinyl) benzamide 5g**

The general procedure **(1)** was followed using (4-((4-fluoropipieridine-1-yl)methyl)phenyl boronic acid to give **5g** as a white solid in 79.7% yield. **^{1}H NMR** (400 MHz, MeOD) δ 8.25 (dd, *J* = 7.9, 1.0 Hz, 1H), 8.05 (d, *J* = 7.1 Hz, 1H), 8.00 (s, 1H), 7.90 (dt, *J* = 7.6, 1.1 Hz, 1H), 7.75 (dt, *J* = 7.6, 1.0 Hz, 1H), 7.67 (m, 3H), 7.46 (m, 4H), 4.68 (m, 1H), 3.60 (s, 2H), 2.99 (s, 3H), 2.56 (m, 4H), 1.90 (m, 4H). **^{13}C NMR** (101 MHz, MeOD) δ 165.3, 147.0, 141.6, 139.8, 138.6, 136.6, 132.4, 132.4, 130.7, 129.9 (2C), 129.0, 127.5(2C), 126.6, 123.6, 123.1, 119.5, 119.2, 88.8. (d, $^{1}J_{CF}$ = 179.8 Hz, 1C), 62.1, 49.0 (d, $^{3}J_{CF}$ = 5.4 Hz, 2C), 43.8, 30.8 (d, $^{2}J_{CF}$ = 19.8 Hz, 2C). **Elemental analysis** (%) found: C, 68.12; H, 5.99; N, 5.98%. $C_{26}H_{27}FN_2O_2S$ requires C, 69.31; H, 6.03; N, 6.22%; **ES+HRMS** *m/z* 451.1844 $[M+H]^+$ $C_{26}H_{27}FN_2O_2S$ requires 450.18.

Preparation of benzo[*d*]isothiazol-3(2*H*)-ones 8-12

General procedure (2)

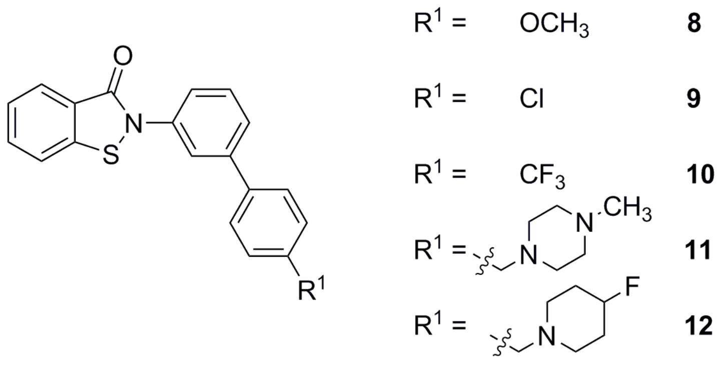

Thionyl chloride (1.3 eq) was added dropwise to a stirring solution of the required biphenyl benzamide (**5c-g**) (1.0 eq) in dichloromethane (15-20ml) under a N_2 atmosphere. The solution was heated to reflux at 50°C for 1.5 hours. Solvent and excess thionyl chloride were removed under reduced pressure and the crude products purified by flash column chromatography.

Preparation of **2-(4'-methoxy-[1,1'-biphenyl]-3-yl)benzo[*d*]isothiazol-3(2*H*)-one 8**

The general procedure **(2)** was followed using **5c** (0.56 mmol) to give **8** as a white-yellow crystalline solid in 58.4% yield. **^{1}H NMR** (400 MHz, DMSO) δ 8.08 (d, *J* = 8.1 Hz, 1H), 7.98 (d, *J* = 7.7 Hz, 1H), 7.95 (d, *J* = 1.7 Hz, 1H), 7.78 (dt, *J* = 8.2, 1.1 Hz, 1H), 7.67 (d, *J* = 8.8 Hz, 2H), 7.65 – 7.56 (m, 3H), 7.53 (t, *J* = 7.8 Hz, 1H), 7.07 (d, *J* = 8.8 Hz, 2H) and 3.82 (s, 3H); **^{13}C NMR** (101 MHz, DMSO) δ 163.9, 159.8, 141.6, 140.6, 138.2, 133.1, 132.0, 130.5, 128.4 (2C), 126.6, 126.5, 125.3, 124.8, 123.1, 122.7, 122.4, 115.0 (2C) and 55.7; **Elemental analysis** (%) found: C, 71.95; H, 4.47; N, 4.41; S, 9.77%. $C_{20}H_{15}NO_2S$ requires C, 72.05; H, 4.54; N, 4.20; S, 9.62%; **ES+HRMS** *m/z* 356.0712 $[M+Na]^+$ $C_{20}H_{15}NO_2S$ requires 333.08.

Preparation of **2-(4'-chloro-[1,1'-biphenyl]-3-yl)benzo[*d*]isothiazol-3(2*H*)-one 9**

The general procedure **(2)** was followed using **5d** (0.46 mmol) to give **9** as a white crystalline solid in 52.2% yield. **^{1}H NMR** (500 MHz, DMSO) δ 8.08 (dt, *J* = 8.1, 0.8 Hz, 1H), 8.02 (t, *J* = 2.1, 1H), 7.98 (ddd, *J* = 7.8, 1.3, 0.7 Hz, 1H), 7.79 (dt, *J* = 8.4, 1.3 Hz, 1H), 7.75 (dt, *J* = 8.7, 2.1 Hz, 2H), 7.71 – 7.67 (m, 2H), 7.64 (t, *J* = 7.9 Hz, 1H), 7.57 (dt, *J* = 8.7, 2.4 Hz, 2H), 7.53 (dt, *J* = 8.0, 1.0 Hz, 1H); **^{13}C NMR** (101 MHz, DMSO) δ 163.9, 140.6, 140.6, 138.5, 138.3, 133.4, 133.2, 130.7, 129.5 (2C), 129.1 (2C), 126.7, 126.6, 125.8, 124.7, 124.2, 123.2, 122.4. **Elemental analysis** (%) found: C, 67.03; H, 3.62; N, 4.13; S, 8.91%. $C_{19}H_{12}ClNOS$ requires C, 67.55; H, 3.58; N, 4.15; S, 9.49%; **ES+HRMS** *m/z* 360.0215 $[M+Na]^+$ $C_{19}H_{12}ClNOS$ requires 337.03.

Preparation of 2-(4'-(trifluoromethyl)-[1,1'-biphenyl]-3-yl)benzo[*d*]isothiazol-3(2*H*)-one 10

The general procedure **(2)** was followed using **5e** (0.43 mmol) to give **10** as a white crystalline solid in 73.6% yield. **^{1}H NMR** (500 MHz, DMSO) δ 8.11 – 8.07 (m, 2H), 7.99 (ddd, *J* = 7.8, 1.3, 0.7 Hz, 1H), 7.97 (d, *J* = 8.2 Hz, 2H), 7.87 (d, *J* = 8.2 Hz, 2H), 7.79 (dt, *J* = 8.3, 1.3 Hz, 1H), 7.78 – 7.74 (m, 2H), 7.69 (t, *J* = 8.0 Hz, 1H), 7.54 (ddd, *J* = 8.0, 1.0 Hz,1H); **^{13}C NMR** (101 MHz, DMSO) δ 163.5, 143.7, 140.6, 140.3, 138.4, 133.2, 130.8, 128.8 (d, $^2J_{CF}$ = 31.7 Hz, 1C), 128.2 (2C), 126.7, 126.6, 126.4 (d, $^3J_{CF}$ = 3.7 Hz, 2C), 126.1, 124.8 (d, $^1J_{CF}$ = 272.0 Hz, 1C), 124.8, 124.7, 123.6, 122.5; **Elemental analysis** (%) found: C, 62.68; H, 3.22; N, 3.19%. $C_{20}H_{12}F_3NOS$ requires C, 64.68; H, 3.26; N, 3.77; S, 8.63%; **ES+HRMS** *m/z* 394.0492 $[M+Na]^+$ $C_{20}H_{12}F_3NOS$ requires 371.06.

Preparation of **2-(4'-((4-methylpiperazin-1-yl) methyl)-[1,1'-biphenyl]-3-yl) benzo[*d*]isothiazol-3(2*H*)-one 11**

The general procedure **(2)** was followed using **5f** (0.549 mmol) to give **11** as an off-white solid in 58.0% yield. **^{1}H NMR** (400 MHz, MeOD) δ 8.07 (d, *J* = 7.9 Hz, 1H), 7.96 (s, 1H), 7.89 (d, *J* = 8.1 Hz, 1H), 7.79 (t, *J* = 7.5 Hz, 1H), 7.69-7.62 (m, 5H), 7.55 (t, *J* = 7.6 Hz, 1H), 7.47 (d, *J* = 7.6 Hz, 2H), 3.61 (s, 2H), 2.90-2.22 (br s, 8H), 2.33 (s, 3H); **^{13}C NMR** (101 MHz, MeOD) δ 164.8, 142.3, 140.8, 139.0, 137.4, 136.9, 132.6, 129.9 (2C), 129.7, 126.7 (2C), 126.1, 125.9, 125.9, 124.2, 123.6, 123.3, 120.8, 61.9, 54.2 (2C), 52.0 (2C), 44.5; **ES+HRMS** *m/z* 416.1810 $[M+H]^+$ $C_{25}H_{25}N_3OS$ requires 415.17.

Preparation of **2-(4'-((4-fluoropiperidin-1-yl)methyl)-[1,1'-biphenyl]-3-yl) benzo[*d*]isothiazol-3(2*H*)-one 12**

The general procedure **(2)** was followed using **5g** (0.44 mmol) to give **12** as an off-white fine crystalline solid in 70.0% yield. **^{1}H NMR** (400 MHz, DMSO) δ 8.08 (d, *J* = 8.1 Hz, 1H), 8.02-7.95 (m, 2H), 7.78 (t, *J* = 7.7 Hz, 1H), 7.71-7.59 (m, 5H), 7.53 (t, *J* = 7.6 Hz, 1H), 7.43 (d, *J* = 7.8 Hz), 4.69 (d, *J* = 50.9 Hz, 1H), 3.52 (s, 2H), 2.60-2.25 (m, 4H), 1.95-1.85 (m, 4H); **^{13}C NMR** (101 MHz, DMSO) δ 163.9, 141.7, 140.6, 138.8, 138.3, 138.2, 133.2, 130.6, 130.0 (2C), 127.1 (2C), 126.7, 126.5, 125.7, 124.8, 123.7, 123.1, 122.4, 89.0 (d, $^1J_{CF}$ = 169.8 Hz, 1C), 61.91, 49.53 (d, $^3J_{CF}$ = 6.0 Hz, 2C), 31.63 (d, $^2J_{CF}$ = 19.0 Hz, 2C); **Elemental analysis** (%) found: C, 71.47; H, 5.56; N, 6.62%.

$C_{25}H_{23}FN_2OS$ requires C, 71.75; H, 5.54; N, 6.69; S, 7.66%; **ES+HRMS** *m/z* 419.1603 $[M+H]^+$ $C_{25}H_{23}FN_2OS$ requires 418.15.

Preparation of **(4-(morpholinomethyl)phenyl) boronic acid 6a**

Morpholine (5.56 mmol, 2.0 eq) and *N,N*-diisopropylethylamine (6.98 mmol, 2.5 eq) were added to a stirring solution of 4-methylbromophenyl boronic acid (2.79 mmol) in anhydrous THF (25 ml) under a N_2 atmosphere. The solution was stirred at room temperature for 18 hours and then quenched with brine (30 ml) and extracted into EtOAc (3 x 30 ml). The combined organic extracts were dried over $MgSO_4$, filtered and concentrated under reduced pressure to give **6a** as a pale orange solid in 87.0% yield which was used as crude in the following step. **^{1}H NMR** (400 MHz, MeOD) δ 7.50 (s, 2H), 7.21 (d, *J* = 7.9 Hz, 2H), 3.58 (t, *J* = 4.6 Hz, 4H), 3.43 (s, 2H), 2.37 (br s, 4H).

Preparation of **4'-(morpholinomethyl)-[1,1'-biphenyl]-3-amine 6b**

To a solution of K_2CO_3 (6.20 mmol, 3.3 eq), **6a** (2.25 mmol, 1.2 eq) and $Pd(PPh_3)_4$ (0.0047 mmol, 0.025 eq) in water (5 ml), 3-bromoaniline (1.88 mmol, 1.0 eq) was added. The stirring reaction was evacuated and purged with N_2 before the addition of anhydrous THF (10 ml) and heating to 80°C for 22 hours. The solution was quenched with brine (50 ml), extracted into EtOAc (3 x 35 ml) and the combined organic extracts dried over $MgSO_4$ before concentrating under reduced pressure. The crude product was purified by flash column chromatography to give **6b** as a pale yellow-brown oil in 91.0% yield. **^{1}H NMR** (400 MHz, $CDCl_3$) δ 7.52 (d, *J* = 8.2 Hz, 2H), 7.37 (d, *J* = 8.2 Hz, 2H), 7.22 (t, *J* = 7.8 Hz, 1H), 6.99 (d, *J* = 8.0 Hz, 1H), 6.90 (t, *J* = 2.0 Hz, 1H), 6.68 (ddd, *J* = 7.9, 2.3, 0.8 Hz, 1H), 3.73 (t, *J* = 4.6 Hz, 4H), 3.53 (s, 2H), 2.48 (m, *J* = 4.2 Hz, 4H); **^{13}C NMR** (101 MHz, MeOD) δ 147.7 141.6, 140.7, 135.7, 129.6 (2C), 129.1, 126.4 (2C), 116.6, 114.3, 113.6, 66.3 (2C), 62.6, 53.2 (2C); **CI+HRMS** *m/z* 269.1650 $[M+H]^+$ $C_{17}H_{20}N_2O$ requires 268.16.

Preparation of **2-(chlorothio)-benzoyl chloride**

Thionyl chloride (22.8 mmol, 7.0 eq) and *N*,*N*-dimethylformamide (3 drops) were added to a solution of 2,2'-dithiobenzoic acid (3.26 mmol, 1.0 eq) in anhydrous dichloroethane (25 ml) under a N_2 atmosphere at room temperature. The stirring solution was heated to reflux at 80°C for 1.5 hours. Sulfuryl chloride (4.24 mmol, 1.3 eq) was added to the cooled solution at 25°C before heating the solution to 50°C for 45 minutes. Solvent, excess thionyl chloride and excess sulfuryl chloride were removed under reduced pressure to generate 2-(chlorothio)-benzoyl chloride, a yellow-green solid as pure product in quantitative yield. **^{1}H NMR** (400 MHz, $CDCl_3$) δ 8.34 (dd, *J* = 8.0, 1.2 Hz, 1H), 7.93 (dd, *J* = 8.2, 0.4 Hz, 1H), 7.77 (dt, *J* = 8.5, 1.3 Hz, 1H), 7.42 (dt, *J* = 8.0, 0.8 Hz, 1H); **^{13}C NMR** (101 MHz, $CDCl_3$) δ 169.5, 146.1, 135.8, 134.7, 127.4, 125.8, 124.4.

Preparation of **2-(4'-(morpholinomethyl)-[1,1'-biphenyl]-3-yl)benzo[*d*]isothiazol-3(2*H*)-one 13**

To a solution of **6b** (1.59 mmol, 1.0 eq) in dichloromethane (10 ml/mmol), 2-(chlorothio)-benzoyl chloride (1.9 mmol, 1.2 eq) was added at 0°C under a N_2 atmosphere. Triethylamine (5.24 mmol, 3.3 eq) was added to the solution which was allowed to warm to room temperature and stirred for 20 hours. The solution was quenched with brine (40 ml), extracted into EtOAc (3 x 50 ml) and the combined organic extracts dried over $MgSO_4$ before concentrating under reduced pressure. The crude product was purified by flash column chromatography to give **13** as a yellow crystalline solid in 79.0% yield. **^{1}H NMR** (400 MHz, MeOD) δ 8.05 (d, *J* = 7.9 Hz, 1H), 7.94 (t, *J* = 1.7 Hz, 1H), 7.87 (d, *J* = 8.2 Hz, 1H), 7.77 (dt, *J* = 7.2, 1.1 Hz, 1H), 7.68-7.62 (m, 4H), 7.59 (d, *J* = 7.6 Hz, 1H), 7.54 (dt, *J* = 7.9, 0.8 Hz, 1H), 7.46 (d, *J* = 8.2 Hz, 2H), 3.71 (t, *J* = 4.7 Hz, 4H), 3.57 (s, 2H), 2.49 (br s, 4H); **^{13}C NMR** (101 MHz, MeOD) δ 164.8, 142.2, 140.7, 138.9, 137.4, 136.8, 132.6, 129.9 (2C), 129.7, 126.7 (2C), 126.1, 125.8, 125.8, 124.2, 123.5, 123.3, 120.7, 66.3 (2C), 62.5, 53.2 (2C); **Elemental analysis** (%) found: C, 71.34; H, 5.57; N, 6.78; S, 7.64%. $C_{24}H_{22}N_2O_2S$ requires C, 71.62; H, 5.51; N, 6.96; S, 7.97%; **ES+HRMS** *m/z* 403.1491 $[M+H]^+$ $C_{24}H_{22}N_2O_2S$ requires 402.14.

Biology

Crystallization

Following nickel affinity purification, *Ec*IspD-A14C was further purified by gel chromatography using a HiLoad 16/60 Superdex 200pg column (GE Healthcare) equilibrated with 10 mM Tris (pH 8.0) and 150 mM NaCl. The fractions containing *Ec*IspD-A14C were pooled and concentrated to 15 mg/mL using an Amicon centrifugal filter. The protein sample was pre-incubated on ice with 5 mM final concentration of compound **8**. The protein crystallized in 0.2 M calcium acetate, 0.1 M HEPES (pH 7.5), 10% PEG 8000. Crystals were flash-frozen under liquid nitrogen for data collection. X-ray data were collected from a single crystal using a wavelength of 1 Å at synchrotron beamline 4.2.2 of the Advanced Light Source in Berkeley, CA. Data were processed with XDS and the coordinates from PDB ID: 1VGT[7] were used as a search model for phase determination by molecular replacement using PHENIX. Automatic model rebuilding was performed in PHENIX. Subsequent iterated manual building/rebuilding and refinement of models were performed using Coot and PHENIX, respectively. The structure validation server MolProbity was used to monitor refinement. A complete summary of final crystallographic refinement statistics are given in Supplementary Table T2. Figures were generated and rendered in PyMOL.

Supplementary references

1. Verdonk, M. L., Cole, J. C., Hartshorn, M. J., Murray, C. W. & Taylor, R. D. Improved protein-ligand docking using GOLD. *Proteins Struct. Funct. Genet.* **52,** 609–623 (2003).
2. Moore, J. L., Taylor, S. M. & Soloshonok, V. A. An efficient and operationally convenient general synthesis of tertiary amines by direct alkylation of secondary amines with alkyl halides in the presence of Huenig’s base. *Ark. (Gainesville, FL, United States)* 287–292 (2005). doi:10.3998/ark.5550190.0006.624
3. Aprile, S., Del Grosso, E. & Grosa, G. In vitro metabolism study of 2-isopropyl-9H-thioxanthen-9-one (2-ITX) in rat and human: evidence for the formation of an epoxide metabolite. *Xenobiotica* **41,** 212–225 (2011).
4. Pietka-Ottlik, M., Potaczek, P., Piasecki, E. & Mlochowski, J. Crucial role of selenium in the virucidal activity of benzisoselenazol-3(2H)-ones and related

diselenides. *Molecules* **15,** 8214–8228 (2010).

5. Baggaley, K. H. *et al.* Inhibitors of blood platelet aggregation. Effects of some 1,2-benzisothiazol-3-ones on platelet responsiveness to adenosine diphosphate and collagen. *J. Med. Chem.* **28,** 1661–1667 (1985).
6. Richard, S. B. *et al.* Structure of 4-diphosphocytidyl-2-C- methylerythritol synthetase involved in mevalonate- independent isoprenoid biosynthesis. *Nat. Struct. Biol.* **8,** 641–648 (2001).
7. Badger, J. *et al.* Structural analysis of a set of proteins resulting from a bacterial genomics project. *Proteins Struct. Funct. Bioinforma.* **60,** 787–796 (2005).
8. Kemp, L. E., Bond, C. S. & Hunter, W. N. Structure of a tetragonal crystal form of Escherichia coli 2-C-methyl-D-erythritol 4-phosphate cytidylyltransferase. *Acta Crystallogr. Sect. D Biol. Crystallogr.* **D59,** 607–610 (2003).
9. Behnen, J. *et al.* Experimental and Computational Active Site Mapping as a Starting Point to Fragment-Based Lead Discovery. *ChemMedChem* **7,** 248–261 (2012).
